# Effect of ready-to-use therapeutic foods on time to recovery among children with severe acute malnutrition in Ethiopia: a prospective cohort study

**DOI:** 10.1186/s12887-023-04168-x

**Published:** 2023-07-05

**Authors:** Arsema Abebe, Yilkal Simachew, Tefera Darge Delbiso

**Affiliations:** 1 Department of Public Health Nutrition and Dietetics, School of Public Health, Addis Ababa University, Addis Ababa, Ethiopia; 2 School of Public Health, College of Medicine and Health Science, Hawassa University, Hawassa, Ethiopia

**Keywords:** RUTF, Severe acute malnutrition, Recovery time, Sidama region, Ethiopia

## Abstract

**Background:**

The therapeutic feeding unit (TFU) provides comprehensive inpatient clinical care for children suffering from severe acute malnutrition (SAM) in three stages: stabilization, transition, and rehabilitation. During the transitional and rehabilitation phases, children receive either F-100 or ready-to-use therapeutic food (RUTF). Although both promote weight gain, RUTF is more energy dense than F-100. There is limited and contrasting evidence regarding their effect on recovery time. Therefore, this study aimed to assess the effect of RUTF on time to recovery among SAM children aged 6–59 months admitted to the TFU in Ethiopia.

**Methods:**

Health Facility-based prospective cohort study was conducted among 476 children treated in three hospitals and four health centers in the Sidama region from September 2021 to January 2022. A structured questionnaire adopted from the Ethiopian national protocol for the management of SAM was used for data collection. Data were entered into EpiData version 3.1 and exported to SPSS version 20 for analysis. The Kaplan-Meir curve and log-rank test were used to compare time to recovery between children who received RUTF and F-100. Multivariable Cox proportional hazard analysis was conducted to assess the association between time to recovery and the type of therapeutic food, controlling for the confounding variables.

**Results:**

The median recovery time was significantly shorter in children receiving RUTF (7 days; 95% CI: 6.62–7.38) compared to F-100 (10 days; 95% CI: 8.94–11.06). Children below 24 months (AHR = 0.54, 95% CI: 0.42–0.69), dehydrated (AHR = 1.34, 95% CI: 1.07–1.75), edematous malnutrition (AHR = 1.29, 95% CI: 1.03–1.61), and anemic (AHR = 2.57, 95% CI: 1.90–3.48) during admission were associated with time to recovery.

**Conclusions:**

Children who received RUTF recovered faster than children who received F-100. Administering RUTF to children below 24 months, who present with anemia and dehydration can improve their recovery rate and shorten their stay in the health facility.

**Supplementary Information:**

The online version contains supplementary material available at 10.1186/s12887-023-04168-x.

## Introduction

Acute malnutrition results from multiple factors, such as reduced dietary intake, increased body requirement, poor absorption, excessive nutrient loss, and medical illnesses [1]. It poses a greater risk of mortality and morbidity from common childhood illnesses and compromises future adulthood health by restricting age-appropriate physical and mental growth. Severe acute malnutrition (SAM) is characterized by the presence of bilateral pitting edema of the nutritional origin or severe wasting (weight-for-height/length z-score (WHZ) below − 3 of the World Health Organization (WHO) growth standard or mid-upper arm circumference (MUAC) < 11.5 cm) in children 6–59 months old [2].

In 2020, SAM affected 13.6 million children worldwide: Africa and Asia share more than 97% of the total burden [3]. According to the Ethiopian Demographic and Health Survey (EDHS) report, wasting and severe wasting is estimated to be 7% and 1% among 0–59 month age children, respectively [4]. SAM is the primary diagnosis in 20% of pediatric hospital admissions in Ethiopia [5]. Based on the annual regional report of 2017, in the Southern region of Ethiopia, where this study was conducted, 42,856 children were diagnosed with SAM, and 15% were complicated SAM cases [6].

According to the SPHERE standards, children should be recovered from SAM within 28 days [7]. However, the recovery time might be prolonged beyond the recommended day due to a combination of factors. A prolonged stay has clinical, social, and economic consequences. It increases the risk of nosocomial infection, emotional disturbance, and long-term behavioral difficulties among children [8, 9]. This, in turn, incurs direct costs for families and institutions and indirect costs for society. A cost-effectiveness study in Ethiopia showed that the institutional cost per child treated was $262.62 in the therapeutic feeding unit (TFU). Moreover, the mother/caregiver is usually required to stay with her child, which is disadvantageous for other family members, commonly for siblings left at home due to less attention, care, and follow-up [9].

The Community Based Management of Acute Malnutrition (CMAM) recommends two SAM management approaches. Children who pass the appetite test and have no medical complications will be treated as outpatients. In contrast, those with one or more medical complications and who failed the appetite test will be treated in the TFU [10]. The TFU provides inpatient clinical therapy for children with complicated SAM using three phases: stabilization, transition, and rehabilitation. The stabilization phase focused on stabilizing the child’s condition by re-feeding with F-75 low protein, low energy formula and treating medical complications. During the transitional and rehabilitation phase, the therapeutic food changed either to ready-to-use therapeutic food (RUTF) (Plumpy’Nut) or F-100 to recover lost weight and catch up with optimal growth.

Both the RUTF (Plumpy’Nut) and F-100 are high-energy, high-protein, and micronutrient-dense therapeutic foods. The RUTF is a paste made from peanuts, while F-100 is a milk-based formula. RUTF offers the malnourished child the same nutrient intake as F-100, with the addition of 10–14 mg/100 g of iron [11]. These therapeutic foods have the same nutrition-to-energy ratio, but RUTF is five times denser in energy than F-100. RUTF is obtained by replacing part of the dried skim milk used in the F-100 formula with peanut butter [12]. Several studies in Ethiopia and different African countries documented predictors of time to recovery [13–18]. However, studies focused on therapeutic foods on time to recovery are limited, and the findings are inconsistent [15, 16, 19]. In a pilot study from India [20] and a randomized controlled trial from Senegal [21], children treated with RUTF had better average weight gain and shorter recovery time. In contrast, another study conducted in India found F-100 to be more effective in promoting short recovery time than RUTF [22]. Therefore, this study aimed to assess the effect of RUTF on time to recovery among SAM children treated at the TFU.

## Methods and materials

### Study design, setting and population

A health facility-based prospective cohort study was conducted among children aged 6–59 months admitted at TFU of selected health facilities from September 2021 to January 2022 in the Sidama region, Ethiopia. Sidama region is one of Ethiopia’s ten regional states, located 273 km south of Addis Ababa, the capital city of Ethiopia. The region has 137 health centers and 17 hospitals (13 primary, 3 general, and one tertiary hospital). The pediatric department in these health institutions is among the main inpatient department where children with complicated SAM are treated.

All children (6–59 months) admitted to the TFU of the selected health facilities in Sidama region with a diagnosis of SAM were the source population. All eligible children from identified health facilities during the study period were the study population.

### Inclusion and exclusion criteria

Children aged 6–59 months with SAM admitted to TFU program during the study period and had entered the transitional phase were included in the study. Children who failed to enter the transitional phase and were re-admitted or relapsed were excluded. Similarly, Children who presented with chronic medical conditions such as HIV/AIDS, CHF, and TB were excluded since their time to recovery is affected by the disease condition.

### Sample size and sampling procedure

To determine sufficient sample size and to find statistically significant effects, power calculations are recommended. Assuming a power of 80%; an alpha value of 5%; the equal proportion of recovery among children taking RUTF and F-100; based on findings from a previous study [15] where the proportion of children recovered was 97.3% and 90.8% for those children who took RUTF and F-100, respectively; and 15% loss to follow-up; the total sample size is calculated to be 476 children (238 RUTF and 238 F-100).

**Fig. 1:**
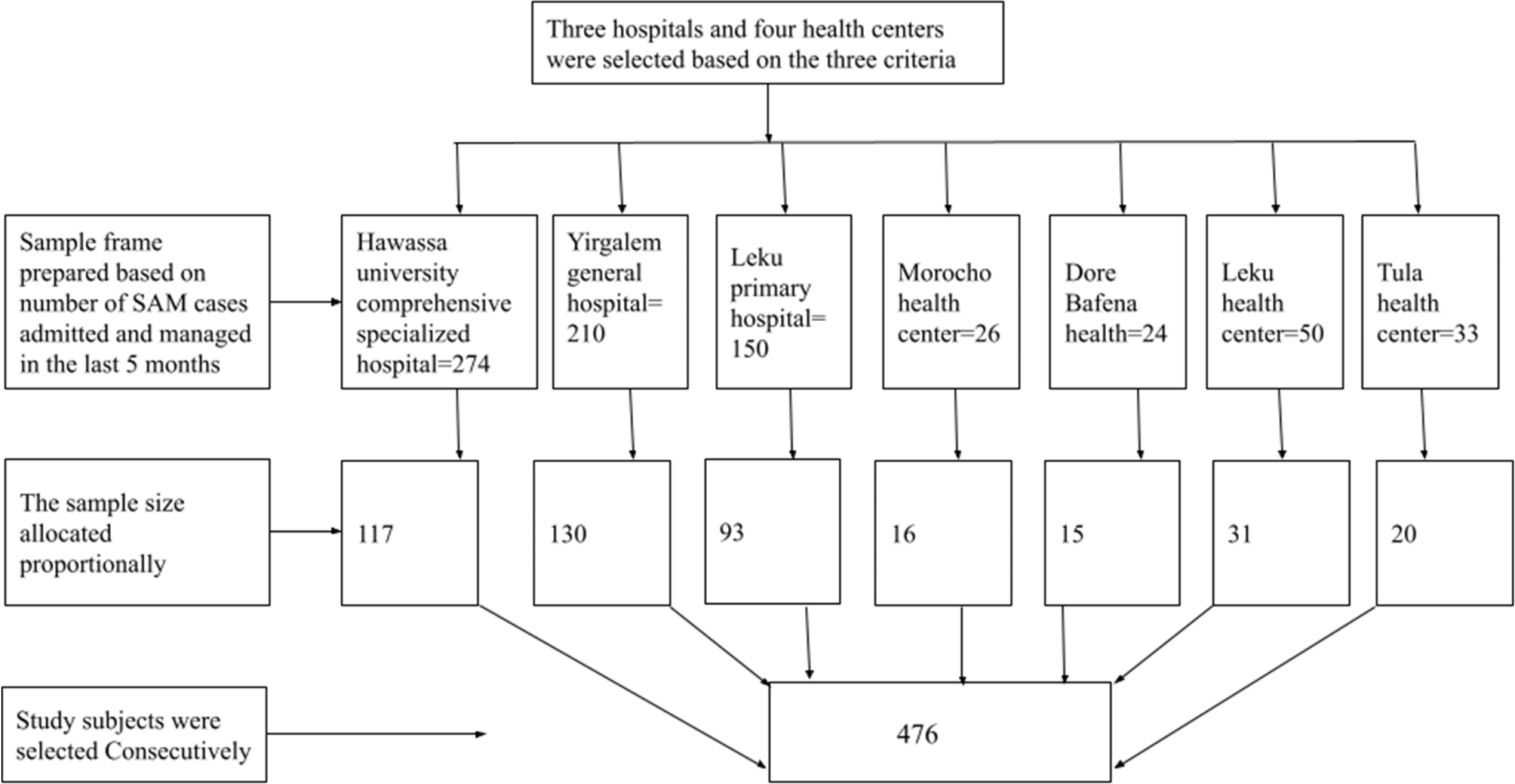
Diagrammatic presentation of the sampling procedure

Of the 137 health centers and 17 hospitals in the region, 68 health centers and all hospitals are providing SAM management service. We selected three hospitals (namely, Hawassa university comprehensive specialized hospital, Yirgalem general hospital, and Leku primary hospital) and four health centers (namely, Morocho health center, Dore Bafano health center, Leku health center, and Tula health center) that satisfy the following three conditions. Those health facilities managing SAM children using either RUTF or F-100 during the transition phase (as some facilities only managed with F-100), admitting and managing SAM children until they were fully recovered (as some facilities referred children to health posts for outpatient therapeutic program once their medical condition was stabilized), and health facilities with high patient flow. Each health facility’s sampling frame was prepared based on the number of SAM cases admitted and treated in the last five months. After determining the average number of admissions for each health facility, the sample size was proportionally allocated according to their size. Then participants were selected consecutively (Fig. 1).

### Study variables

The outcome variable of the study is the time to recovery from SAM treatment. It was calculated by subtracting the date of admission to the transitional phase from the date of discharge and calculating the total number of days the child stayed in the facility. Children who died during the follow-up, did not respond to the treatment, were transferred to other facilities, and did not recover within the follow-up period were considered censored cases.

### Exposure variable

An appetite test was performed during the presentation, and the children were screened for medical complications. Those children fulfilling the admission criteria for inpatient management were admitted directly to the stabilization phase. After they were treated for complications and their clinical condition stabilized (return of appetite, loss of edema, and no indication for intravenous line or nasogastric tubes), they transferred to the transitional phase. During this phase appetite test was done for every child by providing RUTF. A child who passed the test without an allergic reaction was given RUTF throughout the transitional and rehabilitation phase. These children were considered as an exposure group. While those children who were given the milk-based diet F-100 were considered the non-exposure group.

### Confounding variables

The confounding variables include socio-demographic variables (age, sex, and residence of the child), the presenting symptoms, comorbid illnesses, immunization status, medications and nutritional supplements. A full examination, including the grade of edema, dehydration status, skin changes, and vital signs (respiratory rate, pulse rate, and temperature) was performed by the data collectors. Stool examination and hematocrit count were checked from the laboratory report in the medical history sheet.

### Data collection procedures and quality

Data was collected using a structured questionnaire adopted from the Ethiopian national protocol for the management of SAM [12]. The questionnaire used for collecting the data is provided as a supporting file with this manuscript (Additional file 1). During admission, data on the socio-demographic variables (age, sex, and residence of the child), the presenting symptoms, and immunization status were captured. The data collectors performed a full examination including, the grade of edema, dehydration status, skin changes, and vital signs (respiratory rate, pulse rate, and temperature). Stool examination, hematocrit count, and HIV serostatus were checked based on their history.

Daily clinical assessments, including monitoring of vital signs, grade of edema, appetite, amount of feeding, and clinical signs, such as vomiting, diarrhea, and cough through all phases, were performed for each child. Body weight was measured using a digital scale to the nearest 100 g. Length/height was measured using an infant length board/portable height board. MUAC was measured once a week using the color-coded tape meter to the nearest 1 mm. Anthropometric z-scores was computed for WHZ.

The data collection team comprises qualified nurses working in the TFU with previous experience engaging in similar assignments: seven data collectors and three supervisors. Intensive three days of training was given to the data collection team on the objectives, the questionnaire, procedures, and research ethics. The research questionnaire and procedures were pretested on 5% of the total sample at Loke health center, which is not part of the actual study. Ambiguous questions were revised accordingly. The supervisors and the principal investigator monitored the data collection process and the collected data daily. Any issues in the collected data were flagged early, and feedback and necessary measures were taken.

### Data processing and analysis

The collected data were coded and entered into Epi Data version 3.1 and exported to SPSS version 20 for analysis. The data cleaning was carried out to ensure the data quality. Descriptive analysis was carried out to describe the patient’s baseline characteristics using numbers (proportion), median (Inter-Quartile Range – IQR), and graphs. To compare time to recovery between children who received RUTF and F-100, we used the Kaplan-Meir curve and log-rank test. We conducted bivariate Cox proportional hazard analysis to identify the bivariate association between time to recovery and the exposure variable (type of therapeutic food) and the confounding variables. Confounding variables that showed a significant association with time to recovery at < 0.25 level of significance were selected for the multivariable Cox proportional hazard regression. The proportional hazards assumptions were assessed graphically by log minus log survival curve. Variables with p < 0.05 in the final model were considered statistically significant. Then, the hazard ratio (HR) with its 95% Confidence Interval (CI) was reported for variables in the final model.

## Results

### Socio-demographic and baseline characteristics

A total of 476 SAM children were enrolled in the study, making the response rate 100%. Children were included in the follow-up after they completed the stabilization phase. Of the total study subjects in the cohort, 247(51.9%) were male, 232 (48.7%) were below two years of age, 351 (73.7%) were rural residents, 307 (64.5%) were fully vaccinated for their age, and 331(69.5%) were self-referred. The median (IQR) age of the children taking RUTF and F-100 were 26 (23, 37) months and 14 (12, 24) months, respectively, indicating that older children take RUTF. Two hundred eighty of the SAM cases (58.8%) were admitted with wasting, and the rest 196 (41.2%) with edema (Table 1).

**Table 1:** Socio-demographic and baseline characteristics of children 6–59 months of age admitted to TFU Sidama region, Ethiopia, from September 2021-January 2022 **(N = 476)**

Characteristics	Categories	Children taking RUTF	Children taking F100	Total
Sex	Male	130 (54.6%)	117 (49.2%)	247 (51.9%)
	Female	108 (45.4%)	121 (50.8%)	229 (48.1%)
Age	< 24 month	62 (26.1%)	170 (71.4%)	232 (48.7% )
	≥ 24 month	176 (73.9%)	68 (28.6%)	244 (51.3%)
Residence	Urban	70 (29.4%)	55 (23.1%)	125 (26.3% )
	Rural	168 (70.6%)	183 (76.9%)	351 (73.7%)
Source of referral	Self-referral	175 (73.5% )	156 (65.5% )	331(69.6%)
	Other facilities	38 (15.9%)	45 (18.9%)	83 (17.4%)
	Community	25 (10.6%)	37 (15.6%)	62 (13%)
Immunization	Fully vaccinated	189 (79.4%)	118 (49.6%)	307 (64.5%)
	Partially vaccinated	27 (11.3%)	68 (28.6% )	95 (20.0%)
	Unvaccinated	22 (9.3%)	52 (21.8%)	74 (15.5%)
Appetite test	Failed	234 (98.3%)	231 (97.0%)	465 (97.7%)
	Passed	4 (1.7%)	7 (3.0%)	11 (2.3%)
Admission category	Edema	99 (41.6%)	97 (40.8%)	196 (41.2%)
	Wasted	139 (58.4%)	141 (59.2%)	280 (58.8%)
WFH	< 70%	183 (76.9%)	100 (42%)	283 (59.5%)
	> 70%	55 (23.1%)	138 (58%)	193 (40.5%)

### Co-morbid illness during admission

A total of 399 (83.8%) children had at least one type of comorbidity during admission. From the total study subjects, 207(43.5%) had pneumonia, 197(41.4%) had diarrheal disease, 179(37.6%) had vomiting, and 109 (22.9%) had anemia (Table 2).

**Table 2:** Distribution of medical comorbidities among SAM children admitted at TFU in Sidama region, Ethiopia, from September 2021-Janurary 2022

Variable	Categories	Children taking RUTF	Children taking F100	Total (n = 476)
Pneumonia	Yes	130 (54.6%)	77 (32.4%)	207 (43.5%)
	No	108 (45.4%)	161 (77.6% )	269 (56.5%)
Diarrhea	Yes	118 (49.6% )	79 (33.2%)	197 (41.4%)
	No	120 (50.4%)	159 (66.8%)	279 (58.6%)
Dehydration	Yes	63 (26.5%)	58 (24.4%)	121 (25.4%)
	No	175 (73.5%)	180 (75.6%)	355 (74.6%)
Malaria	Yes	17 (7.1%)	28 (11.8%)	45 (9.5%)
	No	221 (92.9%)	210 (88.2%)	431 (90.5%)
Sepsis	Yes	19 (79.8%)	17 (71.4%)	36 (7.6%)
	No	219 (2.2%)	221 (28.6%)	440 (92.4%)
Anemia	Yes	51 (21.4%)	58 (24.4%)	109 (22.9%)
	No	187 (88.6%)	180 (75.6%)	367 (77.1%)

### Medications and nutritional supplements

From the total study subjects, 80.9% of admitted children were given IV antibiotics, such as ampicillin, gentamycin, ceftriaxone, and vancomycin. Regarding the provision of supplementations, (28.9%) of children taking RUTF and (42.4%) of children taking F-100 received vitamin A. In addition, 76.5% of children taking RUTF and 66% of children taking F-100 had taken folic acid. Among the total eligible children to take deworming drugs, 85.1% took either Albendazole/Mebendazole (Table 3).

**Table 3:** Medications and nutritional supplements given for children 6–59 months of age admitted at TFU Sidama region, Ethiopia, from September 2021-January 2022. **(n = 476)**

Treatment given	Categories	Children taking RUTF	Children taking F100	Total
IV antibiotic	Yes	193 (81.1%)	192 (80.7%)	385 (80.9%)
	No	45 (18.9%)	46 (19.3%)	91 (19.1%)
Vitamin A	Yes	69 (28.9%)	101 (42.4%)	170 (35.7%)
	No	169 (71.1%)	137 (57.6%)	306 (64.3%)
Folic acid	Yes	182 (76.5%)	157 (66%)	329 (69.1%)
	No	56 (23.5%)	81 (44%)	147 (30.9%)
Deworming drug	Yes	100 (42%)	66 (27.7%)	166 (34.9%)
	No	138 (58%)	172 (82.3%)	310 (65.1%)
Blood transfusion	Yes	12 (5%)	14 (5.9%)	26 (5.5%)
	No	226 (95%)	224 (94.1%)	450 (94.5%)
NG tube	Yes	13 (5.5%)	28 (11.8%)	41 (8.6%)
	No	225 (94.5%)	210 (88.2%)	435 (91.4%)

### Treatment outcome

SAM children who had completed the stabilization phase and entered the transitional phase were followed prospectively. At the end of the follow up, 340 (71.4%) children recovered and 22 (4.6%) died from the total study subjects. The remaining 6.5%, 3.8%, 4.6%, and 9% ended in defaulter, medical transfer, non-respondent, and transfer out, respectively.

**Fig. 2:**
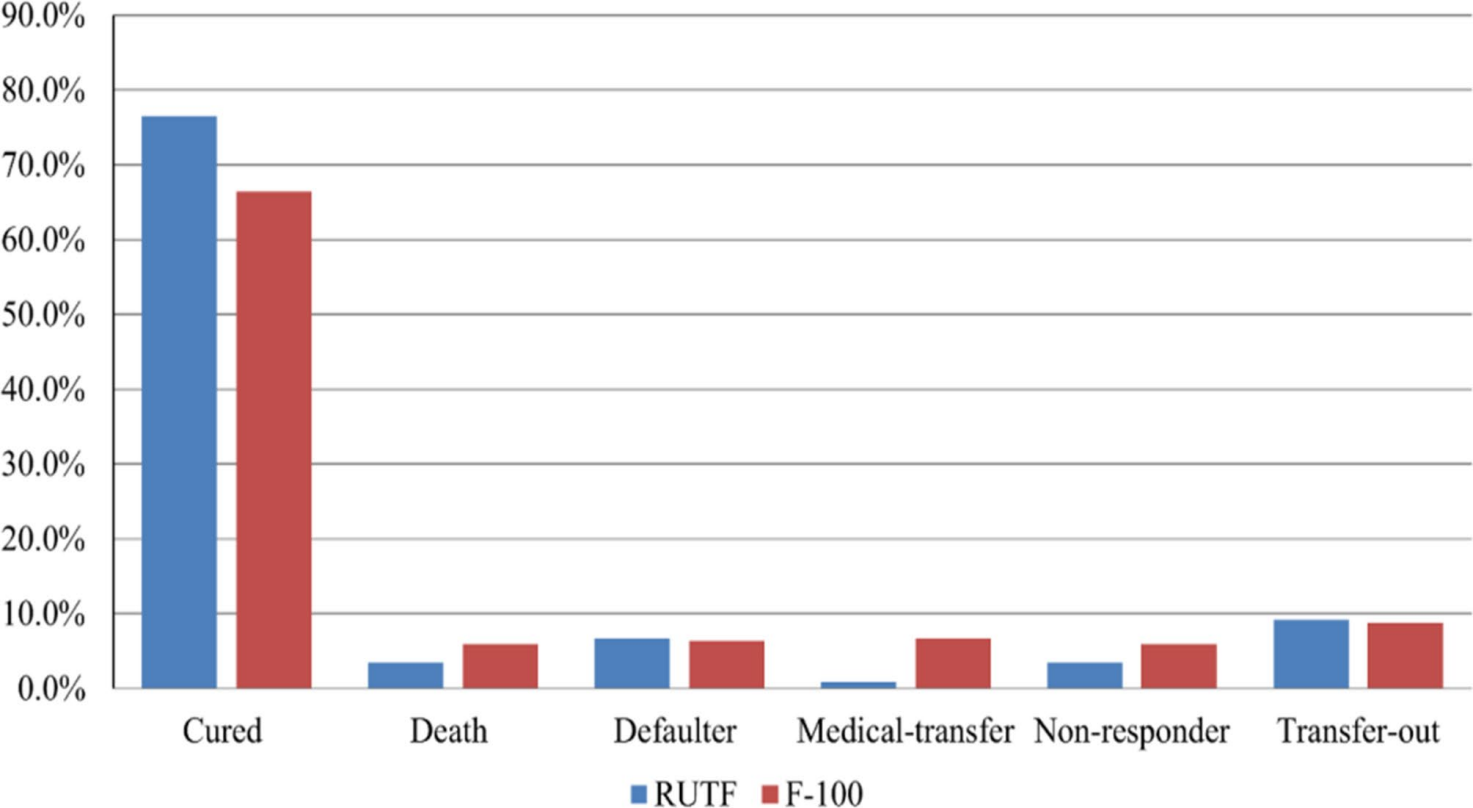
Comparing treatment outcome between children taking F-100 (n = 238) and RUTF (n = 238)

The recovery rate (76.5%) and defaulter rate (6.7%) were relatively high among children taking RUTF, while the death rate (5.9%) and non-respondent rate (5.8%) were high among children taking F-100 therapeutic food (Fig. 2).

### Time to recovery and the Kaplan-Meier survival curve

**Fig. 3:**
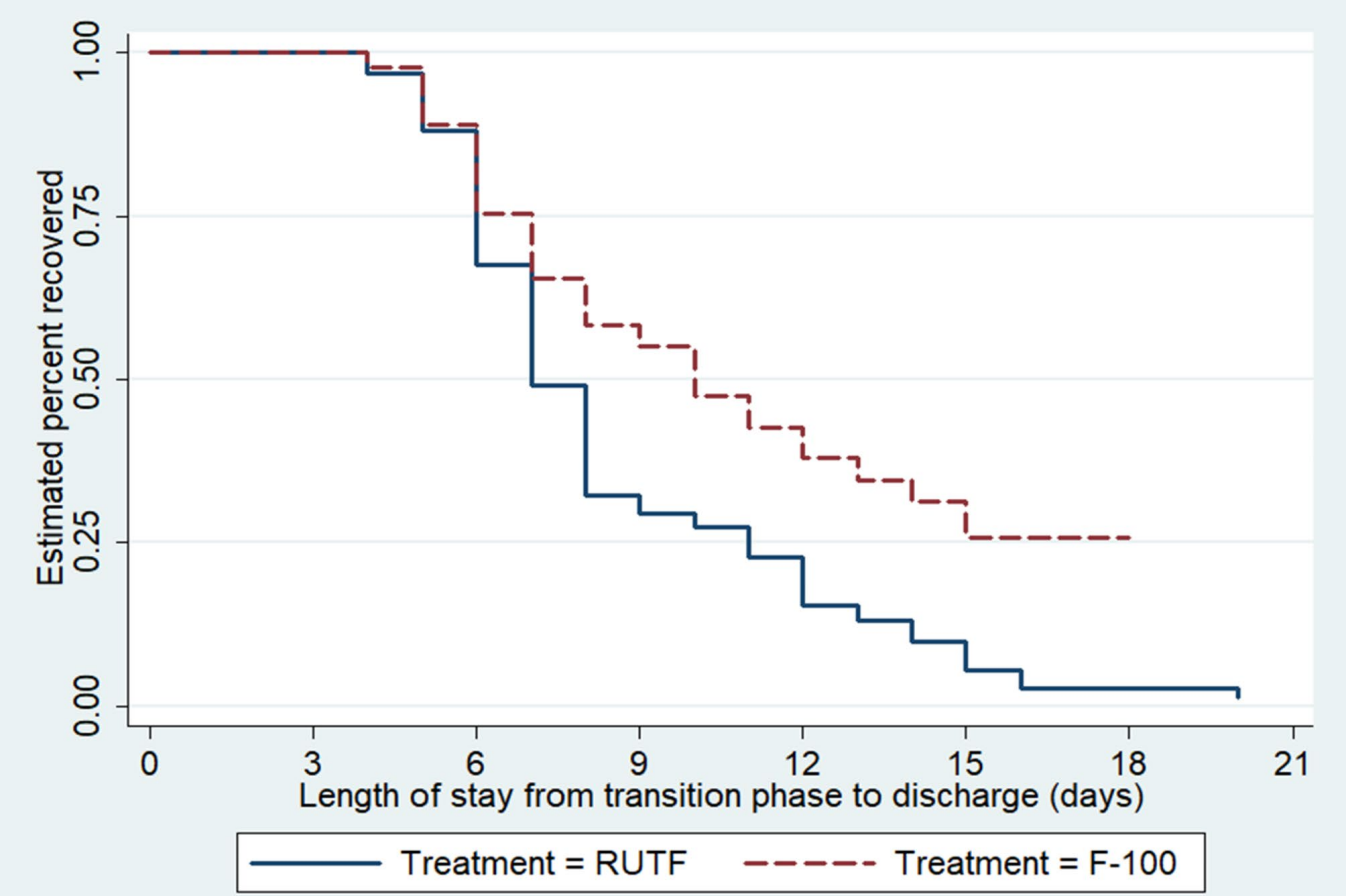
Kaplan-Meier survival curves comparing recovery time between children taking F-100 (n = 238) and RUTF (n = 238)

The median recovery time for the entire cohort was 8 days (95% CI: 7.36–8.64). Kaplan**-**Meier curves with log-rank test show a significant difference in the median recovery time between children taking RUTF (7 days; 95%CI: 6.62–7.38) and children taking F-100 (10 days; 95% CI: 8.94–11.06) with p-value < 0.001 (Fig. 3).

### Factors associated with time to recovery of SAM children

Initially, bivariate cox regression analysis was performed to identify the bivariate association between time to recovery and the exposure variable (type of therapeutic food) and confounding variables (anthropometry during admission (WHZ, MUAC), type of presentation of SAM (wasting and edematous malnutrition), presence of medical complications, and nutritional supplements intake). Based on the result, the type of therapeutic food, age of the child, type of presentation of SAM, folic acid supplementation, anemia, dehydration, and sepsis were significantly associated with recovery time at p < 0.25.

Multivariable cox regression was performed for the above variables after ascertaining the validity of the model assumptions and adjustment. On the multivariable cox regression, type of therapeutic food, age, type of SAM, dehydration, and anemia were found to be independent predictors of recovery time (p < 0.05). After completing the stabilization phase, those children who received RUTF were 1.3 times more likely to recover earlier as compared to children who received F-100 (AHR = 1.30; 95% CI = 1.02–1.66).

Compared to children above 24 months, children below 24 months were 46% less likely to recover faster (AHR = 0.54; 95% CI = 0.42–0.69). Based on the type of SAM, children who were admitted with edema were 1.2 times more likely to recover earlier than children with wasting (AHR = 1.29; 95% CI = 1.03–1.61). Regarding medical illnesses, children who were admitted without dehydration (AHR = 1.37; 95% CI = 1.07–1.75) and anemia (AHR = 2.57; 95% CI = 1.90–3.48) recovered early compared to their counterparts with these conditions (Table 4).

**Table 4:** Multivariate cox regression for factors associated with time to recovery among children 6–59 months of age in Sidama region, Ethiopia, from September 2021 to January 2022

Variables	Categories	Outcome		CHR (95% CI)	AHR (95% CI)
		**Recovered**	**Censored**		
Therapeutic food	RUTF	194 (81.5% )	44 (18.5%)	1.77(1.42–2.20)*	**1.30 (1.02–1.66)**
	F-100	146 (61.3%)	92 (38.7%)	1	
Age	<24 month	143 (61.6%)	89 (38.4%)	2.38 (1.90–2.97)*	**0.54 (0.42–0.69)**
	≥ 24 month	197 (80.7%)	47 (19.3%)	1	
Folic acid	Yes	254 (74.9%)	85 (25.1%)	1	
	No	86 (62.8%)	51 (37.2%)	1.28 (1.00-1.63)*	1.25 (0.97–1.62)
Anemia	Yes	61 (56.0%)	48 (44.0%)	1	
	No	279 (76.0%)	88 (24.0%)	2.77 (2.07–3.69)*	**2.57 (1.90–3.48)**
Dehydration	Yes	101 (74.2%)	35 (25.7%)	1	
	No	254 (74.7%)	86 (25.3%)	1.28 (1.00-1.64)*	**1.37 (1.07–1.75)**
Sepsis	Yes	16 (44.4%)	20 (55.6%)	1	
	No	324 (73.6%)	116 (26.3%)	1.57 (0.95–2.60)	0.74 (0.44–1.22)
Diagnosis	Edematous	160 (81.6%)	36 (18.3%)	1.60 (1.29–1.99)*	**1.29 (1.03–1.61)**
	Severe wasting	180 (64.2%)	100 (35.7%)	1	

*P-value less than 0.25 in the bivariate analysis, Bold for variables with p < 0.05 in the multivariate analysis

## Discussion

We found that children taking RUTF had better recovery time than those taking F-100. In addition, child age, type of malnutrition during admission, and medical complication during admission (dehydration and anemia) were independently associated with recovery time.

The median recovery time for children taking RUTF was shorter (7 days; 95% CI: 6.62–7.38) compared to children given F-100 (10 days; 95% CI: 8.94–11.06). This is in line with the study conducted in Senegal [21], India [23], Bahirdar [16], and Woldia in Ethiopia [24]. However, our finding is inconsistent with findings from other studies conducted in India and St Paulo’s specialized hospital in Ethiopia [15, 22]. The reason might arise from the difference in the management approach and looking at the effect of the therapeutic foods starting from the stabilization phase. In addition, compared to children taking F-100, those taking RUTF are relatively older, which might contribute to the better intake of prescribed food, minimal left over and reduced risk of hospital acquired infection.

The overall median recovery time of the entire cohort was 8 days (95% CI: 7.36–8.64) which is within the SPHERE standard and the accepted national standard of the average length of stay for inpatient treatment [7, 11]. This result is lower than studies conducted in Addis Ababa 17 days (95%CI: 16, 19 days), Bahirdar (16 days with SD of 1.76), Gedeo zone (13.8 days with SD of 9.66) and Hawassa (17 days with SD of 7) and, Zambia (22 days with SD of 11.80) [15, 25–28]. This difference might happen since our study assumes recovery time after children complete the stabilization phase and enter the transitional phase.

Regarding treatment outcome, at the end of the follow-up, there was 71.4% recovery, 4.6% death, 6.5% defaulter, and 9% transfer out of cases. In contrast to the death rate, the cure rate was lower than the standard criteria established by the Ethiopian SAM management protocol [12] and the studies conducted in southern Ethiopia 82.4% and Woldia 85% [13, 24]. This discrepancy could be attributable to a relatively high number of defaulters and transfer out of cases in our study. However, the cure rate in our finding is higher than that of similar studies in Ethiopia, such as a study done in Felegehiwot referral hospital 58.4%, North West Ethiopia 65.8%, and a multicenter study in Amhara region 62.1% [18, 26, 29]. This could be because the studies were carried out in referral and general hospital settings, where patient overload and a high burden of comorbidities, such as chronic diseases (CHF, TB), could account for the low recovery rate. The death rate in this study is 4.6% which is within the acceptable minimum SPHERE standard [7]. But, lower than the reports from studies conducted in other parts of Ethiopia, including Woldia (12%) and North West Ethiopia (10.8%) [24, 30]. This might be explained by a low proportion of diarrhea and pneumonia cases in our study. In addition, children enter the transitional phase after complications are resolved and are in better condition, which decreases the risk of death.

Based on the result, children who were admitted with edema were 1.2 times more likely to recover earlier than children admitted with wasting. The average length of stay among wasted children was 10 (± 1.22) days and 8 (± 0.45) days among children with edema. The finding was in line with a study conducted in southern Ethiopia [13]. However, in contrast to the study conducted in the Amhara region, North Shoa Ethiopia, and Malawi [29–31]. This might be due to the difference in the magnitude of comorbidities during the initial phase and adherence to the standard treatment protocol. In addition, in this study, most edematous children are taking RUTF, which promotes fast recovery time by preventing the increased risk of fluid overload, which is a common complication among edematous patients.

Children above 24 months have a short recovery time compared to children below 24 months. This is similar to a study conducted in Aksum and Yekatit’s 12 hospitals [32, 33]. This is due to depressed immunity, increased risk of communicable diseases, and inadequate feeding practices among young children [31]. In addition, age brings a difference in appetite, preference, and intake of the amount of therapeutic food.

Children admitted without dehydration have a shorter recovery time than children who have dehydration. The recovery time was delayed among children who were anemic during admission. This is similar to a finding at Pawi general hospital in northwest Ethiopia [18] and a study in northwest Ethiopia [30] where children without anemia had recovered faster than anemic children. This happens because there is an increased prevalence of infection and increased risk of heart failure among children presented with anemia that end with a prolonged recovery time.

### Conclusions and recommendations

The finding showed that children who received RUTF during the transitional and rehabilitation phases recovered faster compared to children who received F-100: 7 days (95%CI: 6.62–7.38) for RUTF and 10 days (95% CI: 8.94–11.06) for F-100. The overall median recovery time was within the SPHERE standard 8 days (95% CI: 7.36–8.64). However, the recovery rate (71.4%) remains low compared to the standard criteria as per Ethiopia’s 2019 SAM management protocol. Children less than 24 months old, being anemic, dehydrated, and non-edematous were independently associated with prolonged recovery time.

To conclude, health facilities and healthcare professionals should provide RUTF for children who meet the criteria and tolerate it without any allergy. Special attention and care need to be given to children below 24 months who present with anemia, dehydration, and edema to improve the recovery rate and shorten their stay. Future research would benefit from including hospital-related factors, such as quality of service and skill of professionals, which might affect the outcome and recovery time.

## Electronic supplementary material

Below is the link to the electronic supplementary material.

Supplementary Material 1

Supplementary Material 2

## Data Availability

The datasets supporting the conclusions of this article are included within the article.

## Abbreviations

AHR: Adjusted Hazard Ratio
CHD: Congenital Heart Diseases
CHR: Crude Hazard Ratio
CI: Confidence Interval
CMAM: Community Based Management of Malnutrition
EDHS: Ethiopian Demographic and Health Survey
AIDS: Human Immune Deficiency Disease
IQR: Inter Quartile Range
MUAC: Middle-Upper Arm Circumference
OTP: Out Patient Therapeutic Program
RCT: Randomized Control Trial
RUTF: Ready to Use Therapeutic Food
SAM: Severe Acute Malnutrition
SC: Stabilization Center
SD: Standard Deviation
SNNPR: South Nation’s Nationalities and Peoples Region
TFC: Therapeutic Feeding Center
TFU: Therapeutic Feeding Unit
UNICEF: United Nations International Children’s Emergency Fund
WFA: Weight for Age
WFH: Weight for Height
WHO: World Health Organization
