# Supplementary material for: Effect of ready-to-use therapeutic foods on time to recovery among children with severe acute malnutrition in Ethiopia: a prospective cohort study
Source: BMC Pediatr. 2023 Jul 5;23:340. doi: 10.1186/s12887-023-04168-x (PMC10321003; doi:10.1186/s12887-023-04168-x)

## Additional file 1: Questionnaire used for data collection

## Annex 1: Consent Form

**Information Sheet and Consent Form**

Effect of ready to use therapeutic food on time to recovery among children with severe acute malnutrition admitted to therapeutic feeding unit in Sidama region, Ethiopia: A prospective cohort study

General Information for Study Participants

Good morning/afternoon.

My name is Mr. /Ms.……………………. I am postgraduate student of Adiss Ababa University and I am doing research on the effect of ready to use therapeutic food on time to recovery among children with severe acute malnutrition admitted to therapeutic feeding unit. The results of this study could be used to improve the average length of stay of children admitted with severe acute malnutrition at therapeutic feeding units. I am going to follow your children until he/she discharged from the therapeutic feeding unit. From time of admission to discharge, I will ask you some questions regarding the socio-demographic factors, presence of medical complication and also conduct anthropometric measurements (weight, height, MUAC) and take vital signs. I would like to assure you that the information of your child will be kept strictly confidential and will not be shown to other people. In addition, your name will not be used in any document throughout this study. You have been selected at random to participate in this study. Your honest participation will help the researcher complete this study.

May I continue? Yes ……….. No ………..

If you say “yes” sign below

I have been briefly informed about the study and clearly understood the objective of the study. So I here approve my consent with my signature to take part in the study.

Signature ____________Date _____________

If no, please thank the interviewee and stop.

Interviewee’s signatures …………. Date………………….

Name of the principal Investigator: Arsema Abebe

Mobile: 0917100289/0941050775. E-mail: [arsema.abebe21@gmail.com](mailto:arsema.abebe21@gmail.com)

Checked by: Supervisor Name __________ Signature ________Date _________


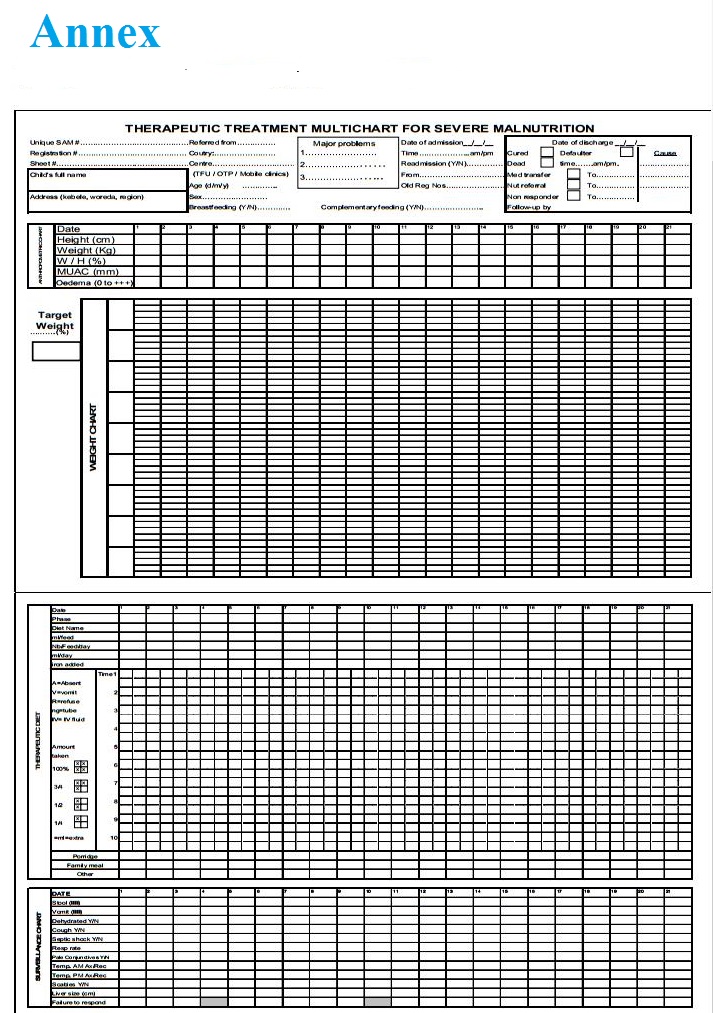


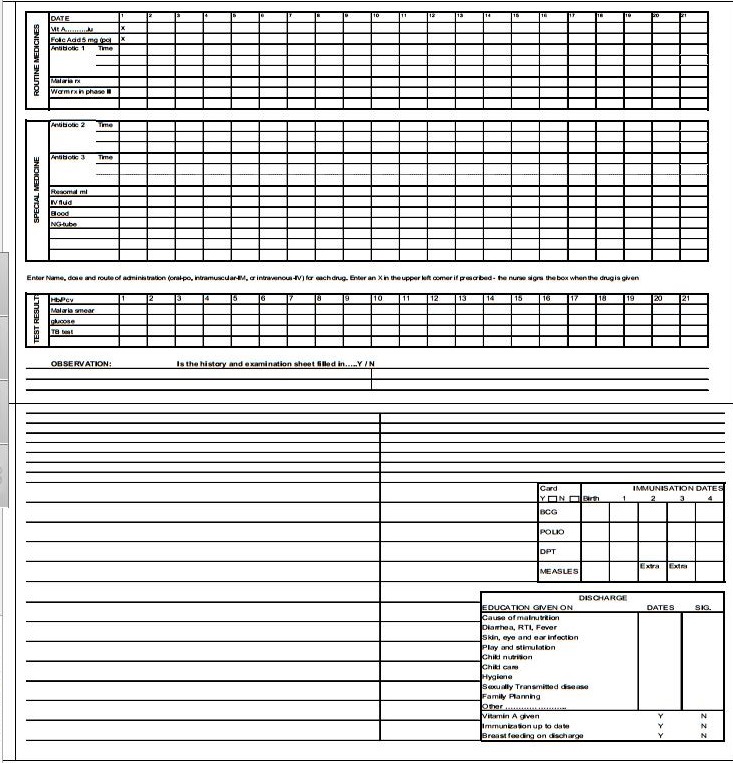

Supplement: Supplementary file 1 — Supplementary Material 1 [file 12887_2023_4168_MOESM1_ESM.docx]
